# 3D Printed Polymeric Hydrogels for Nerve Regeneration

**DOI:** 10.3390/polym10091041

**Published:** 2018-09-19

**Authors:** Binoy Maiti, David Díaz Díaz

**Affiliations:** 1 Institute of Organic Chemistry, University of Regensburg, Universitätstr. 31, 93053 Regensburg, Germany; binoymaiti1@gmail.com; 2 Instituto de Química Avanzada de Cataluña-Consejo Superior de Investigaciones Científicas (IQAC-CSIC), Jordi Girona 18-26, 08034 Barcelona, Spain

**Keywords:** nerve regeneration, 3D printing, polymeric hydrogels

## Abstract

The human nervous system lacks an inherent ability to regenerate its components upon damage or diseased conditions. During the last decade, this has motivated the development of a number of strategies for nerve regeneration. However, most of those approaches have not been used in clinical applications till today. For instance, although biomaterial-based scaffolds have been extensively used for nerve reparation, the lack of more customized structures have hampered their use in vivo. This highlight focuses mainly on how 3D bioprinting technology, using polymeric hydrogels as bio-inks, can be used for the development of new nerve guidance channels or devices for peripheral nerve cell regeneration. In this concise contribution, some of the most recent and representative examples are highlighted to discuss the challenges involved in various aspects of 3D bioprinting for nerve cell regeneration, specifically when using polymeric hydrogels.

## 1. Introduction

Nerve injuries are very common and serious clinical trauma that may cause partial or total loss of motor, sensory, and autonomic functions. Every year more than 200,000 nerve repair procedures are performed by surgeons with a consequent cost of millions of dollars only in the United States [1]. Most commonly, the autograft method (i.e., tissue transplanted from one part of the body to another in the same individual) has been used as first-line therapy for repairing damaged peripheral nerves [2,3,4]. Nevertheless, there are many unavoidable disadvantages associated to the autograft method. For example, it requires an additional secondary surgical site, from where the donor nerve should be taken, while being also a time-consuming and costly process. Moreover, a diameter mismatch between defected nerves and newly grafted nerves, limited the donor sources [5]. In some cases, the autograft method achieves limited success in clinical practices mainly due to immunogenic rejection and disease transmission [6]. In this regard, alternative approaches involving the use of artificial biomaterials have received increasing attention in the field of nerve regeneration. Initially, two-dimensional (2D) models [7] or prefabricated hollow channels filled with polymeric scaffolds have been used to stimulate neurons growth [8]. However, the main disadvantages of 2D models lie on the facts that they do not provide a natural three-dimensional (3D) environment for neuronal cell growth under in vivo conditions. Significant efforts have already been made by numerous research groups to regulate the neuronal growth by using 3D polymeric hydrogel networks [9]. Polysaccharides such as chitosan, ref. [10] alginate, refs. [11,12,13] hyaluronic acid and derivatives [14,15] constitute attractive candidates for in vivo nerve regeneration due to their biocompatible and biodegradable nature. In this sense, the use of biodegradable polymers for constructing nerve guide channels is ideal because it eliminates the need of a second surgery to remove the nerve guide channels from the body to avoid chronic tissue responses or nerve compression. In particular, poly (phosphoester) [16,17], collagen [18,19], polyglycolide [20], collagen and poly-glycolide [21], poly (l-lactide-*co*-glycolide) (PLGA) [22,23], and poly-l-lactic acid/caprolactone [24] are among the most common biodegradable polymers used for this purpose.

Moreover, the evaluation of nerve injuries is not an obvious process, especially when the damage affects several nerves with different lengths and geometries (e.g., branched structures). Different patient anatomies and injury profiles have encouraged researchers to develop more personalized treatments for peripheral nerve injuries. In this case, standard nerve conduits with simpler architectures made from polymeric scaffolds using conventional manufacturing methodologies are not enough to solve this problem because more customized architectures are needed to mimic the same anatomical structure of the damaged nerve. In this context, 3D printing techniques have drawn great attention during the last decade, allowing the preparation of personalized medical devices such as amputee prosthetics, airway splints, and a variety scaffolds for tissue engineering [25]. 3D printing technology helps to fabricate more precise nerve growth channels, providing an open inner structure that enhances the supply of nutrients and nerve growth factors to embedded cells. The toxicity and insufficient mechanical strength of different synthetic materials constitutes two of the most important limitations generally found in 3D printing applications (*vide infra*).

This concise highlight briefly describes how 3D printing of polymeric hydrogels has been used for nerve regeneration, and what are the main limitations that have been found in such processes. Thereafter, the most recent progress in 3D bioprinting for nerve regeneration are also discussed along with a description of the most relevant criteria that must be considered for the selection of suitable hydrogel scaffolds. The description of biomaterial-based hydrogels for nerve regeneration that have not been prepared using 3D printing techniques are described elsewhere [9,26] and are out of the scope of this contribution.

## 2. 3D Printing Technology

In general, 3D printing refers to any process in which a particular material is joined or solidified under computer control to create a 3D object of specific geometry, usually by successively adding material layer-by-layer. Two of the most common technologies for this purpose are the stereolithography (SLA), where photopolymerization is primarily used to produce a solid part from a liquid, and fused deposit modeling (FDM), where the desired part is produced by extruding small beads or streams of material that harden immediately to form layers. The term “3D bioprinting” alludes to the use of 3D printing techniques to combine cells, growth factors, and biomaterials to fabricate biomedical parts that mimic natural tissue features [27].

3D bioprinting is based on three main approaches: Biomimicry, autonomous self-assembly and mini-tissue building blocks [28]. The main objective of the biomimicry approach is to fabricate structures that are identical to those found in natural tissues and organs. For this, it is necessary to understand the microenvironment, the nature of the biological forces in such microenvironment, the specific organization of functional and supporting cell types, solubility factors, and the composition of extracellular matrix. The autonomous self-assembly approach relies on the physical process of embryonic organ development as a model to replicate the tissues of interest. In other words, autonomous self-assembly demands a deeper understanding of the mechanisms involved in the formation of embryonic tissues. Finally, in the mini-tissue approach, small functional components manufactured and arranged into larger framework to build organs and tissues.

From the manufacturing point of view, 3D bioprinting generally follows three steps [29,30]: (1) Pre-bioprinting or creation of the model, (2) bioprinting using the liquid mixture of cells, matrix and nutrients known as bioinks, and (3) post-bioprinting, a final process to create a stable structure from the biological material. Nowadays, sophisticated bioreactor technologies [27] have allowed the rapid maturation of tissues, vascularization of tissues and the ability to survive transplants [30]. Inkjet, laser-assisted, and extrusion printers are the three major types of printers used for 3D bioprinting [29]. Inkjet printers are mainly used in bioprinting for fast and large-scale products, while extrusion printers print cells or hydrogels infused with cells layer-by-layer to create 3D constructs. In addition to just cells, extrusion printers may also use hydrogels infused with cells.

## 3. Recent Reports on 3D Printing Technology for Nerve Regeneration

**Figure 1:**
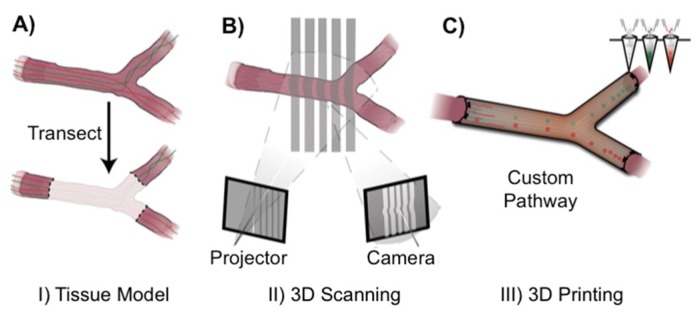
Schematic representation of 3D printing, (**A**) transection of complex nerve (**B**) Imaging of transected nerve (**C**) functionalization of the 3D printed model with physical cues, and path-specific biochemical cues. Adapted with permission from ref. [31]. Copyright 2015, Wiley-VCH.

**Figure 2:**
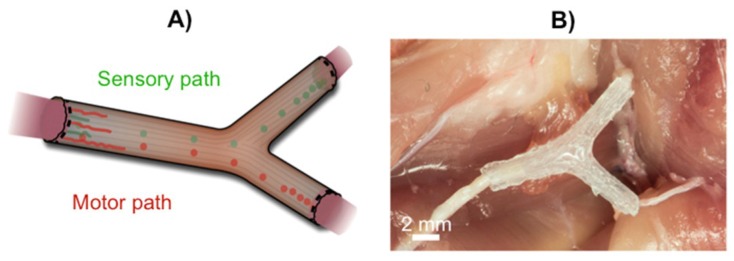
(**A**) Schematic of implanted nerve guide showing bifurcation into sensory and motor nerve paths. (**B**) Photograph of an implanted 3D printed nerve guide prior to suturing. Adapted with permission from ref. [31]. Copyright 2015, Wiley-VCH.

Conventional nerve guidance channels are generally fabricated around tubular structures, and the resultant devices are integrally restricted to linear structures. For more complex anatomical structures and internal biofunctionalization, Johnson and co-workers developed a new 3D printing strategy using silicone as a raw material (Figure 1) [31]. The first step consisted in collecting all the information about the missing nerve pieces and fed those data into the 3D printer. Subsequently, printing of the computational model of the image and simultaneous functionalization with physical cues and path-specific biochemical gradients was carried out (Figure 1) to stimulate the nerve growth in a specific direction. The obtained silicone 3D printed conduit was then inserted into the rat body by surgically grafting it onto the damaged ends of the nerve. This successfully resulted in the regeneration of bifurcated injuries across a 10 mm complex nerve gap in rats (Figure 2) within ca. 10 to 12 weeks. This method provides a mechanism for redeveloping injured nerve plexuses, which is difficult to achieve using conventional nerve guidance channels. Although these results open the door for making different types of nerve regeneration implants more precisely with complex shapes, the main disadvantage of this approach is the use of non-biocompatible silicone, which should be replaced by biodegradable alternatives in future studies.

**Figure 3:**
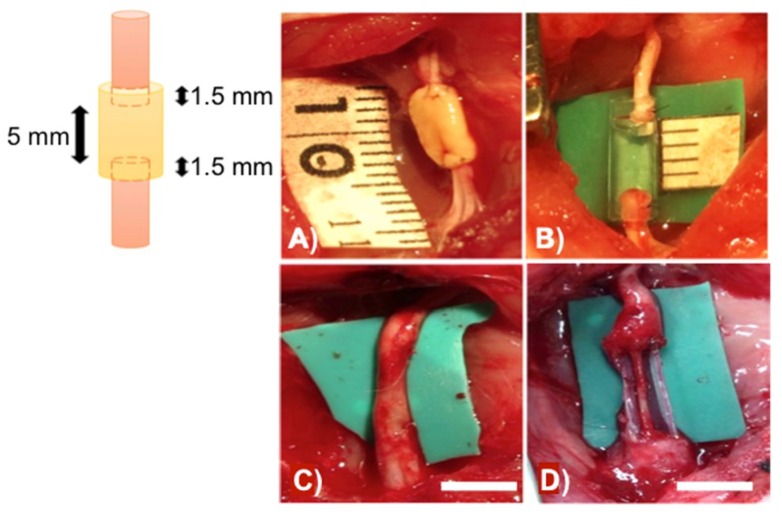
(**A**) Bio 3D conduit was implanted into the nerve defect, and the proximal and distal nerve stumps were secured 1.5 mm into the tube to create a 5-mm interstump gap in the conduit. (**B**) The silicone tube with 8 mm length was implanted in the same procedure. (**C**) Regenerated sciatic nerve eight weeks after surgery in the bio 3D group and (**D**) silicone tube. Scale bar in (**C**,**D**) = 5 mm. Adapted with permission from ref. [32]. Copyright 2017, Public Library of Science.

In many cases, nerve conduits made of synthetic materials may be toxic to the patients, being often associated to various infections. To address these potential problems, Ikeguchi’s team has recently developed a novel approach for nerve regeneration without the use of synthetic material by 3D bioprinting [32]. The authors made scaffold-free bio 3D conduits using human dermal fibroblasts as base material (i.e., a cell generated ECM support). Subsequently, the conduits were tested for nerve regeneration in adult male rats and the results were compared to those obtained using synthetic silicone-based nerve conduits. After 8 weeks of post-surgery, the bio 3D conduits composed entirely of fibroblast cells showed better nerve regeneration ability than the silicone-based nerve conduits (Figure 3). However, further studies are still required to determine the efficiency of 3D bio-conduits in clinical applications. In this sense, the degradation mechanisms of the bio-conduits as well as their mechanical strength and flexibility should be evaluated in detail.

**Figure 4:**
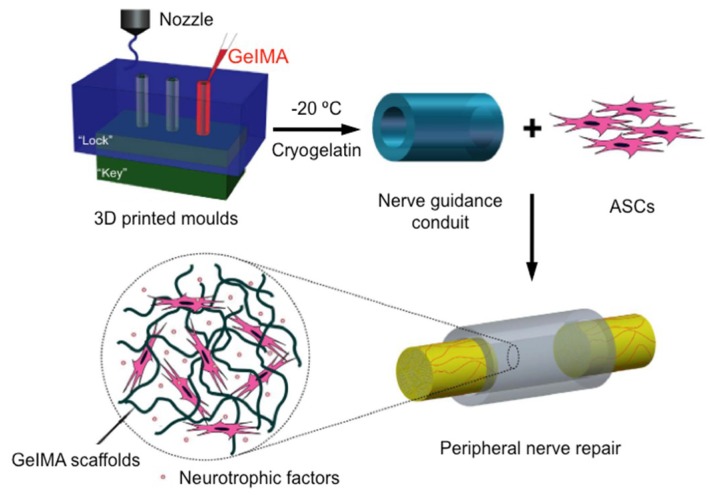
Schematic presentation of the 3D engineered bio-conduit for peripheral nerve regeneration. Adapted with permission from ref. [33]. Copyright 2016, Nature Publishing Group.

By using 3D printing technology Hu and co-workers have also developed an interesting bio-conduit for peripheral nerve regeneration that consist of a cryopolymerized gelatin methacryloyl (cryoGelMA) gel cellularized with adipose-derived stem cells (ASCs) [33]. The cryoGelMA gel was designed into conduits with customized architectures such as multichannels and bifurcated structures by using 3D-printed “lock and key” molds (Figure 4). The main advantage of using cryoGelMA conduit is that it is biodegradable and could be completely degraded in vivo within 2–4 months, eliminating the need of a second removal surgery.

**Figure 5:**
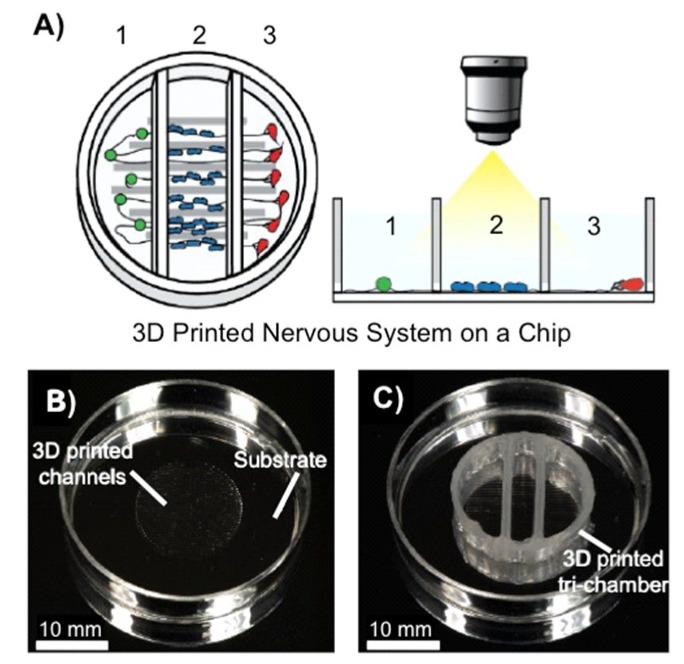
(**A**) Scheme of a representative 3DNSC for peripheral nervous system applications, showing (1) PNS neurons in chamber 1, (2) Schwann cells in chamber 2, and (3) terminal cell junctions in chamber 3. The Schwann cells and the terminal cells interact with the neurons and each other solely via the axonal network. (**B**) Circular pattern of 3D printed silicone microchannels for axonal guidance in the center of a plastic 35 mm dish. (**C**) A 3DNSC showing perpendicular assembly of microchannel and tri-chamber components. Adapted with permission from ref. [34]. Copyright 2016, The Royal Society of Chemistry.

The model nervous system, composed of different biomaterials and cells have different neurological properties. In order to capture different neurological phenomena such as cell signalling, communication, infection, regeneration and degradation, advanced in vitro models are required. Herein, microfluidics, chamber-based technologies, and 3D cell culture models are emerging as the most effective technologies to study different such processes associated with different biomaterials and cells. Recently, McAlpine and co-workers have developed a 3D printed peripheral nervous system on a chip (3DNSC) to study viral infection in the nervous system. The 3D printed system is partitioned into three chambers by dividers and consists of parallel microchannels and a sealant layer. Peripheral neurons are cultured in the first chamber, Schwann cells in the middle chamber, and terminal cell junctions containing successfully formed axon termini and epithelial cells in the third chamber (Figure 5) [34].

## 4. Basic Criteria for Hydrogel Selection

Various nerve conduits for nerve regeneration made from different hydrogels, but not using 3D printing technology, have been reported elsewhere [35,36]. However, in vivo experiments have demonstrated that 60% of those tubes showed similar responses to autografts, while 40% were significantly worse likely due to tube collapse [37]. The development of new suitable and customized 3D printed scaffolds that can direct neuronal growth in the desired direction constitutes a major scientific challenge. Within this context, hydrogels are very attractive candidates due to their high water content, and the fact that they provide a suitable 3D environment for cell growth. In addition, during the gelation process it is possible to incorporate different drug molecules and/or nerve growth factors into the gel matrix for subsequent release. However, there are a few critical aspects that should be considered before using any hydrogel for 3D bioprinting such as sufficient biocompatibility, effective cell adhesion to the gel matrix, and good mechanical stability right after printing and during culture. For example, collagen is a naturally occurring polymer that possesses low toxicity and has been widely used for nerve regeneration [38,39]. However, the mechanical strength of the hydrogel made from collagen is low compared to other synthetic polymers. Thus, in order to increase the mechanical strength and other critical properties such as permeability rate, compressive modulus, cell number, and cell metabolic activity, collagen has been modified with other natural and synthetic polymers [40]. Furthermore, rheological properties such as viscosity, storage modulus, yield stress, and shear thinning play also a key role in 3D bioprinting [41,42,43]. Note that for different printing processes, the values of the above-mentioned parameters are crucial for selecting the optimal gel formulation. Within this context, Table 1 outlines the main properties of the most representative hydrogels reported so far as bioinks for 3D bioprinting. Additionally, the swelling behavior of the hydrogels depends on the crosslinking density. Thus, increasing the crosslinking density in the gel results in lower swelling ratios, thereby reducing the circulation of oxygen and other nutrients that are required for the embedded cells to survive into the 3D hydrogel environment.

## 5. Conclusions and Future Perspectives

Significant progress has been made over the past few decades in the area of nerve regeneration and target reinnervation [62]. However, full recovery of complex injuries containing mixed nerves at a bifurcation is extremely challenged due to imprecise customized shape of nerve guidance channels. Moreover, nerve regeneration across gaps greater than 30 mm remains a critical challenge, particularly for patients who suffer multiple injuries due to trauma. Very recently, 3D printing and additive manufacturing technology have successfully evolved into printing more specified customized scaffolds for peripheral nerve repair. In this context, polymer chemists and material scientists are called to develop unique biodegradable hydrogel scaffolds that will fulfill all biological and mechanical requirements for developing tissue-engineered constructs via automated 3D printing processes. Advanced 3D in vitro mathematical models are required to mimic faster and more precisely the anatomical structure and physiological properties of specific nerves. Moreover, better preclinical models and optimized in vitro–in vivo translatability have been identified in numerous studies as major needs. In this sense, novel nerve-on-a chip technologies constitute a promising approach for developing more translatable in vitro models [63].

It should be considered that a successful regenerative process is established not only at the injured nerve, but also on distal sites that are also affected upon peripheral injuries (e.g., muscle atrophy, sensory receptor degeneration). Thus, a deeper understanding of the biology and biochemistry associated to nerve damage is of utmost importance to maximize the interaction between injured nerves and 3D printed scaffolds. Moreover, continuous improvements in the accuracy of 3D printers and optimized bioinks are expected in the next few years [64].

Further advances from preclinical animal models in research laboratories to human clinical trials will require not only a significant reduction of financial costs, but also intense interdisciplinary cooperations between polymer chemists, material scientists, physicists, computationalists, pharmacists, and physicians.

## Figures and Tables

**Table 1:** Properties of different hydrogels used in 3D bioprinting.

Hydrogel	Applied Material Oncentration (*w*/*v*)	Mechanical Roperties ^a^	Viscosity (Pa/s)	Gelation Method ^b^	Bioprinting Technique ^c^	Cell type Density Cells/mL ^d^	Cytocompatibility /Biodegradability	Refs.
Alginate	Alginate/Ca^2+^ 1%–3%/0.5%	Elastic modulus λ = 21.35 kPa	2.9 at a shear rate of 91 s^−1^	IC	Inject	RHECs 500,000	83%/yes	[44,45]
	Sodium alginate 1%	-	0.12 shear rate not reported	IC	Laser-assisted	HUEVCs (Eahy926), 6 × 10^7^ and Rabbit carcinoma cells (B16) 4 × 10^7^	High, day 1/-	[46]
Gelatin	Methacrylate GelMA/gelatin 5%/8%	Young’s modulus Y = 4.85 ± 0.41 kPa	10−100 at a shear rate of 1–500 s^−1^	PC	EB	BMSCs 5.0 × 10^6^	<90%/yes	[47]
	GelMA/alginate/4-arm PEGTA 5%–7%/1%–3%/1%–3%	Compressive moduli = 24.2–50.7 kPa	28−54 at a shear rate of 7.74 s^−1^ 0.08 Pa s^−1^	PC	Inject	HUVECs MSCs 3 × 10^6^	80%–90%, day 7/yes	[48]
	GelMA/alginate/Ca^2+^ 4.5%/1%–4%/0.3–0.6 M	λ = 15 – 55 kPa	0.08 shear rate not reported	PC/IC	EB	HUVECs-	75%, day 5/-	[49]
	GelMA/GelSH & heparin 10%/1%	Compressive moduli = 1 ± 2 kPa	-	Thiol-ene	-	Human articular chondrocytes 15 × 10^6^	74%–86%, week 5/-	[50]
Poly (ethylene glycol)	Dimethacrylate 10%; 20%	Compressive moduli = 395.73 ± 80.40 kPa	-	PC	Inject	Human articular chondrocytes 5 × 10^6^	89%, day 1/-	[51]
	Diacrylate/alginate 20%/12.5%	λ = 5.3 ± 0.9 to 74.6 ± 1.5 kPa	-	PC	EB	PAVIC 20 × 10^6^	ca. 100%, day 21/-	[52]
Hyaluron-ic acid (HA)	Methacrylate (HA-MA)/GelMA 1.5%/-	Storage modulus *G*′ = 80–90 Pa Loss modulus *G*″ = 40 Pa	-	PC	Inject	HepG2 C3A Int-407 NIH 3T3 2.5 × 10^5^	Cell proliferation *p* < 0.05/yes	[53]
	Gelatin-methacrylamide/HA 20%/2.4%	Compressive modulus = 7995 kPa	-	PC	Inject	Chondrocytes 5 × 10^6^	82% ± 8%, day 3/yes	[54]
	HA/hydroxyethyl-methacrylate derivatized-dextran (dex-HEMA) 2%–6%/10%	*G*′ = 10 kPa	70 at a shear rate of 0.1 s^−1^ and >10 at a shear rate <10 s^−1^ for 2% HA and 10% DexHEMA	PC	EB	Chondrocytes-	75%±19%, day 3/yes	[55]
	PEG-tetraacrylate/yaluronic acid 3%–5%/1.5%–2.5%	*G*′ = 100–800 Pa	-	Michael addition	Microcapillary tube-style printing	NIH 3T3; HepG2 C3A; Int 407 25 × 10^6^	ca. 100%, week 4/yes	[56]
	HA/methyl cellulose 0.25%–2.0%/0.5%–9%	*G*′ = 10–1000 Pa	-	Thermal	EB	MSCs-	75%, day 15/-	[57]
	Hyaluronic acid hydrogels grafted with laminin-	-	-	PC	Photopatterned layer-by-layer	Schwann cells-	Cells retained at 36 h/yes (enzymatically)	[58]
p(HPMAm-lac)-PEG-p(HPMAm-lac)	25%–35%	λ = 119 kPa	-	Thermal/PC	EB	Chondrocytes 5.0 × 10^6^	94%, day 1/yes	[59]
Polycaprolactone (PCL)	PCL with gelatin/PEGDA-	Y = 1.43 ± 0.33 mPa	-	PC	Stereolithography and electrospinning	NE-4C NSCs -	Enhancement in cell proliferation, day 5/-	[60]
Polyurethane	Polyurethane with PCL 25%–30%	G′ = 680–4000 Pa	-	Supramolecular (hydrogen bonding)	Fused-deposition manufacturing	NSCs 4 × 10^6^	ca. 100%, day 3/yes	[61]

^a^ Storage modulus = *G*′; loss modulus = *G*″; ^b^ Ionic crosslinking = IC; photocrosslinking = PC; ^c^ Extrusion-based = EB; ^d^ Rat heart endothelial cells = RHECS; bone marrow stem cells = BMSCs; human umbilical vein endothelial cells = HUVECs; human mesenchymal stem cells = MSCs; porcine aortic valve interstitial cells = PAVICs; human hepatoma cells = HepG2 C3A; human intestinal epithelial cells = Int-407; murine fibroblasts = NIH 3T3; neural stem cells = NSCs.
